# Supplementary material for: Circulating neprilysin hypothesis: A new opportunity for sacubitril/valsartan in patients with heart failure and preserved ejection fraction?
Source: PLoS One. 2021 May 14;16(5):e0249674. doi: 10.1371/journal.pone.0249674 (PMC8121351; doi:10.1371/journal.pone.0249674)
Supplement: Attachment — Submitted filename: Response to reviewers-Plos One.docx [file pone.0249674.s003.docx]

**Reviewer #1:**

1- **Hypothesis properly outlined in the Introduction.**

Thank you.

**2- Methods: Were samples collected on patients, stored and then all analyzed with the same assay at once or analyzed over a 10-year period? sNEP is not an assay normally processed in regular hospital laboratories. This needs to be clarified before accepting for publication.**

We appreciate your comment and apologize for the generated doubts. Blood samples were collected and stored at -80°. Afterwards they were processed in two time-periods: 1069 patients in June-July 2014 and the rest in November 2018. We clarify this aspect in the methods section: “…Blood samples were obtained between 09:00 am and 12:00 pm and stored at at -80º and analyzed without previous freeze-thaw cycles. Analyses were performed in two time periods: June-July 2014 in the first 1069 patients and November 2018 in the rest”.

**3.-CV death: defined by the authors. Appropriate follow up and tracking of events/outcomes.**

Thank you.

**4- Results: Can the authors provide the number of patients screened for this study? Unclear how many patients screened – must be clarified prior to publication.**

Thank you for the comment. We clarify this question in the revised version of the manuscript: “Circulating sNEP was measured in 1,428 patients with HF who were consecutively enrolled in the study from May 2006 to February 2016, out of the 1,765 patients who were attended during this period time. No clinical criteria for exclusion were established and only consent and availability of blood sample determined the included patients”.

**5- Table 1: demographics are consistent with previous studies (HFPEF vs. HFREF).**

We agree with the comment.

**6- Hidralazine (Table 1) should be spelled hydralazine**

Done.

**7- Table 2: Data similar to other studies i.e. worse outcomes in patients with HFREF vs. HFPEF.**

In the revised version of the manuscript we added the following sentence in the limitations section: “…Due to the worse prognosis observed, we cannot discard a selection bias in our patients with LVEF >57%”.

**8- Discussion: appropriate comments made on results. Discussion of limitations of testing.**

We appreciate your comment. In the revised version of the manuscript we added the following limitation: “…Technical limitations of the assay should be acknowledged. Only circulating soluble NEP was assessed, but in humans NEP is widely expressed in several organs, and thus the results reported here might underrepresent overall NEP expression and activity. Furthermore, the experimental assay we used for sNEP determination has long incubation times and it is not ready for clinical use. Up to our knowledge there are no marketed immunoassays approved in clinical practice.

Due to the worse prognosis observed, we cannot discard a selection bias in our patients with LVEF >57%..”

**Reviewer #2: Lupon et. al. present results from a large cohort of patients with HF hospitalized over a 10-year period. sNEP is measured and associations are examined among those with and without preserved EF (>=57%) and above and below median of sNEP. The association between sNEP and outcomes in HFpEF patients warrants further external validation. Further, whether only those who have elevated sNEP will benefit from ARNI will require further studies. These limitations are nicely outlined**.

**Additional investigations could consider:**

**-AUC for sNEP in patients with and without pEF.**

Thank you for suggestion. We choose Harrell’s C-statistic because it takes into account time-to-event, although it always gives lower numbers that AUC by logistic regression. In the revised version of the manuscript we added:

“Harrell’s C-statistic for sNEP above/below the median, taking also in consideration age and sex, was 0.730 (0.682-0.779) in patients with LVEF >57% and 0.677 (0.664-0.776) in patients with LVEF ≤57% for the primary composite end-point, and 0.704 (0.643-0.765) and 0.629 (0.603-0.655), respectively, for the secondary composite end-point of cardiovascular death or HF hospitalization)”.

**-Remove either the event-free survival or cumulative incidence as this information is redundant in the figure.**

We apologize for the lack of clarity. As mentioned in the figure legend, left panel (A) represents event-free survival for the composite end-point of all-cause death or HF hospitalization, while right panel (B) represents cumulative incidence of the composite end-point of cardiovascular death or HF hospitalization (with competing risk taken into account in the analysis). We clarify this in the reviewed figure.

**-Add discussion about whether sNEP could be used before HFpEF develops to predict risk for HF**

Thank you for your recommendation. In the revised version of the manuscript we added the following paragraph in the Discussion section:

“Up to or knowledge sNEP levels have not been evaluated for diagnostic purposes in HF. With the hypothesis that higher sNEP levels would correlate with lower natriuretic peptide levels, worse diastolic function, and subsequent clinical incident HFpEF, Reddy et al. [20] performed a population study with 1,536 participants from Olmsted County, Minnesota. The authors found that low sNEP was paradoxically associated with worse diastolic dysfunction and hypertension but not with outcomes, including incident HF over a median of 10.7 years of follow-up.”

[20] Reddy YNV, Iyer SR, Scott CG, Rodeheffer RJ, Bailey K, Jenkins G, et al. [Soluble Neprilysin in the General Population: Clinical Determinants and Its Relationship to Cardiovascular Disease.](about:blank) J Am Heart Assoc. 2019; 8: e012943. doi: 10.1161/JAHA.119.012943.
